# Secondary Metabolites of *Purpureocillium* *lilacinum*

**DOI:** 10.3390/molecules27010018

**Published:** 2021-12-21

**Authors:** Wei Chen, Qiongbo Hu

**Affiliations:** Key Laboratory of Bio-Pesticide Innovation and Application of Guangdong Province, College of Plant Protection, South China Agricultural University, Guangzhou 510642, China; cw@stu.scau.edu.cn

**Keywords:** entomogenous fungi, biosynthesis, biocontrol, nematodes

## Abstract

Fungi can synthesize a wealth of secondary metabolites, which are widely used in the exploration of lead compounds of pharmaceutical or agricultural importance. *Beauveria*, *Metarhizium*, and *Cordyceps* are the most extensively studied fungi in which a large number of biologically active metabolites have been identified. However, relatively little attention has been paid to *Purpureocillium lilacinum*. *P. lilacinum* are soil-habituated fungi that are widely distributed in nature and are very important biocontrol fungi in agriculture, providing good biological control of plant parasitic nematodes and having a significant effect on *Aphidoidea*, *Tetranychus cinnbarinus*, and *Aleyrodidae*. At the same time, it produces secondary metabolites with various biological activities such as anticancer, antimicrobial, and insecticidal. This review attempts to provide a comprehensive overview of the secondary metabolites of *P. lilacinum*, with emphasis on the chemical diversity and biological activity of these secondary metabolites and the biosynthetic pathways, and gives new insight into the secondary metabolites of medical and entomogenous fungi, which is expected to provide a reference for the development of medicine and agrochemicals in the future.

## 1. Introduction

The genus *Purpureocillium* in the Ophiocordycipitaceae family was structured by Luangsa-Ard et al. In 2011, based on the medical importance, the *Purpureocillium lilacinum* was designated as the type species of the *Paecilomyces* genus [1]. This species was nominated as *Penicillium lilacinum* by Thom in 1901, and then it was revised as *Paecilomyces lilacinus* by Samson in 1974 [2]. After comparing the 18S rRNA gene, internal transcribed spacer, and partial translation elongation factor 1-a sequences with *P. lilacinus*, Luangsa-Ard proposed a new genus name *Purpureocillium* and made the new combination *P. lilacinum* in 2011. The fungus was found in a wide range of land and marine environments [3,4,5]. They are often isolated from insects, nematodes, and the rhizosphere of many crops [6,7,8]. The species can grow in a wide range of temperatures from 8 to 38 °C with optimal temperatures of 26–30 °C [3]. It also has a wide pH tolerance and can grow on a variety of substrates [9]. This fungus has promising potential as a biocontrol agent to control crops‘ root-knot nematodes [10,11]. The parasitism of nematodes is that the hyphae directly invade the surface of nematodes’ eggs and then produce appressoria on the surface, which infects the nematodes’ eggs after adsorption. In the process of infection, *P. lilacinum* secretes a variety of enzymes, such as serine protease and chitinase, which can lead to the degradation of protein and chitin components of the nematode epidermis, which is conducive to the invasion of fungi and destruction of cell components. It has been shown that the fermentation filtrate of *P. lilacinum* can inhibit the mycelial growth of the pathogenic fungi *Helminthosporium maydis* and *Fusarium graminearum*, and has a significant inhibitory effect on the spore germination of *Fusarium oxysporum* [12,13]. Currently, there are eight registered pesticide products of *P. lilacinum* in China used to control root-knot nematodes (http://www.chinapesticide.org.cn/hysj/index.jhtml, accessed on 23 February 2021); similar pesticides are also registered in the USA (https://iaspub.epa.gov/apex/pesticides, accessed on 23 February 2021) and European Union (http://www.efsa.europa.eu/, accessed on 23 February 2021). In addition, *P. lilacinum* has been shown to be effective against *Phyllotreta striolata, Thrips palmi,* and predatory mite [14,15]. However, the *P. lilacinum* strains used for biocontrol agents have a high identity with those strains causing infections in humans [16,17].

Secondary metabolites are produced in a certain growth period of plants and microorganisms. They are small molecules with complex chemical structures that are not necessary for growth and reproduction, such as pigments, hormones, toxins, and antibiotics [18]. Fungi are important organisms that produce active secondary metabolites. Different kinds of fungi produce different secondary metabolites. The discovery of fungal secondary metabolites has become an important source of new drugs and pesticides [19]. Fumosorinone was isolated from the *Isaria fumosorosea*, and it is a potential medicine for the treatment of type II diabetes and other associated metabolic syndromes [20]. Diorcinol K, D, and I were isolated from *Aspergillus*, displaying significant antibacterial activities against *Staphylococcus aureus* and methicillin-resistant *S. aureus* [21]. Pyrenocine A was produced by *Paecilomyces* and showed a significant antitrypanosomal activity against *Trypanosoma brucei* [22]. Paeciloxanthone was isolated from the extracts of *Paecilomyces* sp. and showed significant cytotoxicity against HepG2 cell lines [23]. The research on the synthesis and regulation of secondary metabolites is helpful to develop new active compounds and increase the output of active compounds. The common secondary metabolites of fungi are polyketones, nonribosome peptides, sterols, alkaloids, and terpenes.

Several interesting reviews related to various aspects of *Paecilomyces* species have been published. For example, Weng reviewed the risks of *Isaria fumosorosea* (formerly *P. fumosoroseus*) and *I. farinose* (formerly *P. farinosus*) and their mycotoxins, including their structures, bioactivities, and toxicities [24]. Zhang summarized the mycotoxins of *I. cicadae* (formerly *P. cicadae*) and *I. tenuipes* (formerly *P. tenuipes*), as well as their risk evaluation [25]. As far as we know, a comprehensive overview of *P. lilacinum*, with an emphasis on the chemical diversity, relevant biological activities, and biosynthesis of these metabolites, remains untouched. In this paper, according to the published research reports in recent years, we have sorted out the secondary metabolites of *P. lilacinum* and described the sources, chemical structures, and bioactivities of the reported compounds with particular emphasis on their potential use as drug lead compounds and general biosynthesis pathways. We provide a reference for the follow-up study and ensure more secure and efficient use of fungal products.

## 2. Leucinostatins

Leucinostatins is a class of peptide mycotoxins that have some potent effects on liver cells after oral administration, and they were used as both antimicrobial and antitumor agents through interactions with the membrane phospholipids [26].

**Figure 1:**
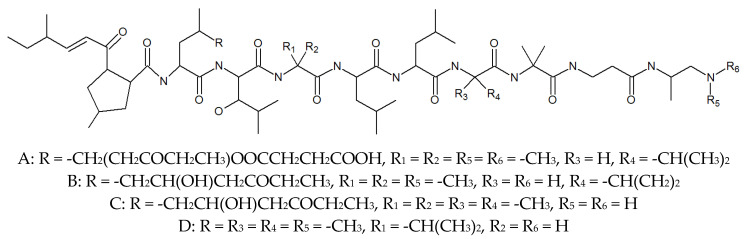
The structures of leucinostatins.

In the early 1970s, Tadashi and his team isolated a new antibiotic from the culture medium of *P. lilacinum*, named leucinostatin (Figure 1, Table 1). It was found that leucinostatin A, B, C, and D were active against yeasts including pathogenic and nonpathogenic strains and filamentous fungi, and were moderately active against Gram-positive bacteria, but more important is its anticancer activity, which is cytotoxic to the HeLa cell culture and inhibits Ehrlich subcutaneous solid tumors and prostate cancer [27,28]. A new report indicated that leucinostatin has a significant antihypertensive effect on rabbit blood pressure, but does not affect the reaction of adrenaline and acetylcholine [29]. It is worth mentioning that leucinostatin is one of the active substances to kill nematodes. Manabu suggested that Leucinostatin A inhibited prostate cancer cell growth through the reduction in insulin-like growth factor I expression in prostate stromal cells [30]. In addition, the use of leucinostatin A-loaded nanospheres could represent a new promising therapeutic system against Candida infection [31]. Leucinostatin Y exhibited the promising importance of the C-terminal of leucinostatins on the preferential cytotoxicity and inhibition of mitochondrial function of cancer cells in the absence of glucose [32]. It is not difficult to see that leucinostatins are potential anticancer compounds, but it should also be noted that the acute toxicities determined by intraperitoneal LD_50_ values for leucinostatins A and B hydrochlorides were found to be 1.8 mg/kg in mice body weight. Thus, it should be evaluated more carefully when it comes to drug use [33,34]. In addition, Yuzuru separated to obtain leucinostatin A, B, C, D, H, and K in *P. lilacinum* and found that the different isolates all produced leucinostatins, which means that they may play an important role not only in the infection process of mycoses in the human case, but also with insects and nematodes [26].

## 3. PK Metabolites

### 3.1. Acremonidins and Acremoxanthones

**Figure 2:**
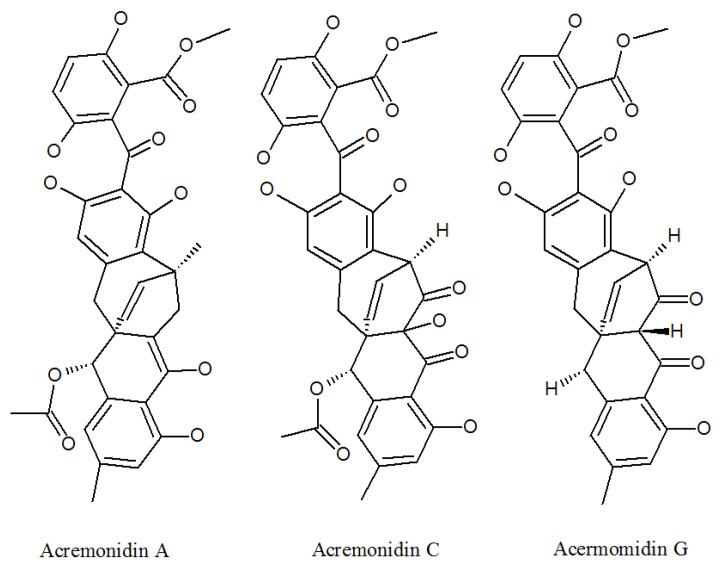
The structures of acremonidins.

**Figure 3:**
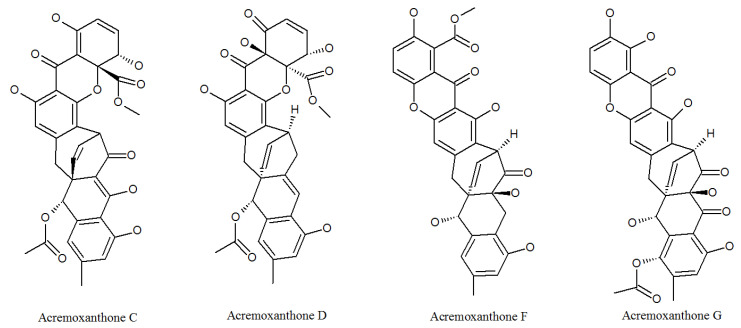
The structures of acremoxanthones.

Acremonidins (Figure 2, Table 1) and acremoxanthones (Figure 3, Table 1) are kinds of polyketides (PKs), which contain alternating carbonyl and methylene groups, usually involved in cell defense or intercellular communication.

Acremoxanthone and acremonidin are xanthone-anthraquinone heterodimers, both calmodulin inhibitors produced by *P. lilacinum* [35]. Both of them exhibited anti-*Bacillus cereus*, antibacterial, antifungal, antiplasmodial, and cytotoxic activity [36]. In addition, acremonidin A and C, and acremoxanthone C and D were found to be moderate for 20 s proteasome inhibitory activity. Acremonidin A and Acremoxanthone C also have high affinity with human calmodulin biosensors. This means that they are also calmodulin inhibitors [37]. In addition, researchers found that acremoxanthone C has anti-oomycete activities [38]. Two new compounds acremoxanthones F and G represented antimalarial activity against the *Plasmodium falciparum* K1 strain, a multidrug-resistant strain [39]. These studies indicate that acremonidins and acremoxanthones all have high medicinal and economic value, but their toxicological effects need to be evaluated in more detail.

### 3.2. Paecilomide

**Figure 4:**
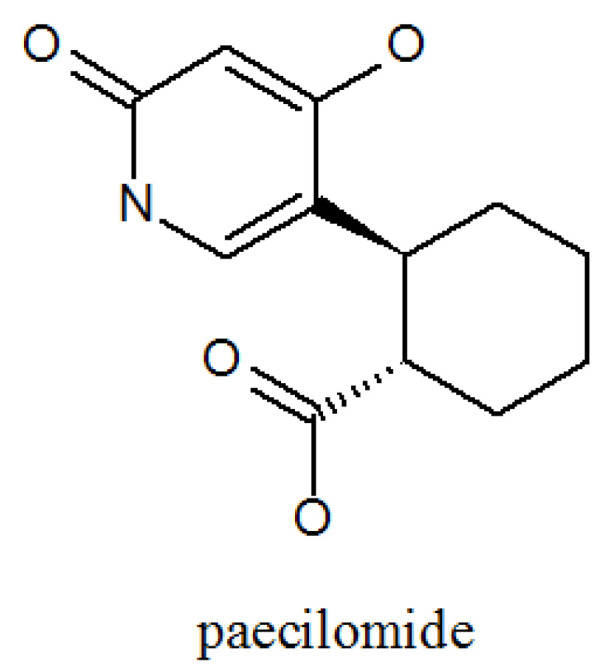
The structure of paecilomide.

Teles found a new α-pyridone Paecilomide (Figure 4, Table 1) by adding salmonella typhimurium to a flask containing *P. lilacinum* in two different concentrations and three different forms at two different stages of the development of *P. lilacinum*. He observed that adding a small amount of inoculum produced a living extract, while adding a large amount of inoculum led to the extraction of active extract, paecilomide [40]. It is an acetylcholinesterase inhibitor [41,42].

### 3.3. Pyrones

**Figure 5:**
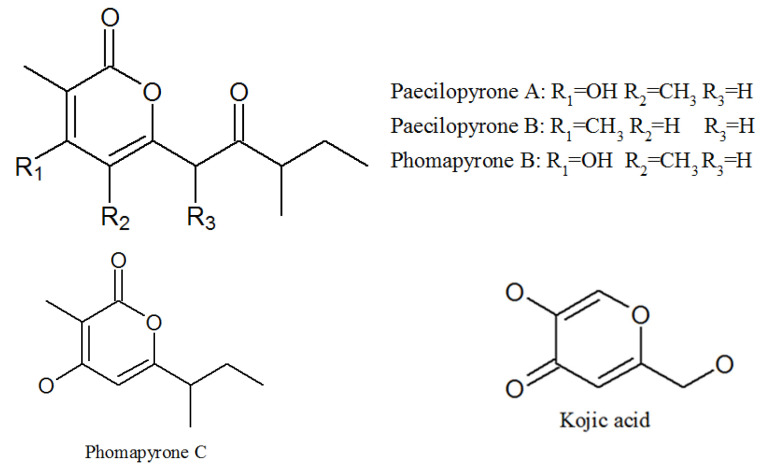
The structures of pyrones.

Elbandy isolated 13 compounds from the sponge-derived fungus *P. lilacinum* in 2009, four of which were α-pyrones: paecilopyrone A, paecilopyrone B, phomapyrone B, and phomapyrone C; and one γ-pyrone: kojic acid (Figure 5, Table 1). Paecilopyrone A and its linear analog paecilopyrone B may be derived from pentaketide; unfortunately, the detailed synthetic pathway is still unclear [43]. Kojic acid showed antibacterial activities and tyrosinase inhibitory activity [44,45].

### 3.4. Phomaligols

**Figure 6:**
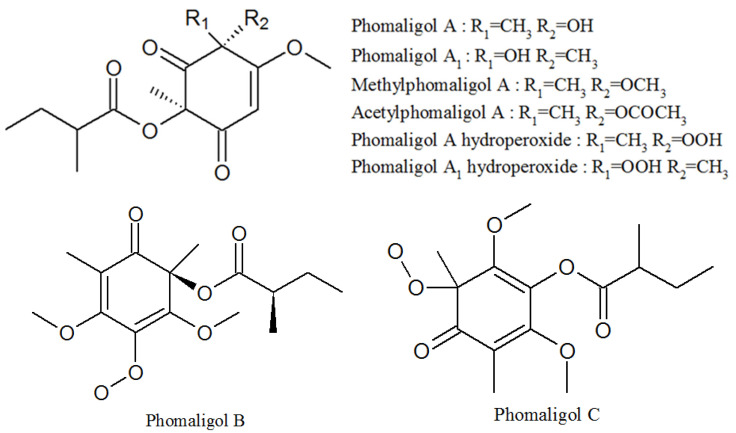
The structures of phomaligols.

Phomaligol A, phomaligol A1, methylphomaligol A, acetylphomaligol A, phomaligol A hydroperoxide, phomaligol A1 hydroperoxide, phomaligol B, and phomaligol C were all isolated from the culture of *P. lilacinum* (Figure 6, Table 1). All of them are yellow oil. Phomaligol A and Methylphomaligol A both showed cytotoxicity against a small panel of human solid tumor cell lines [46]. Phomaligol A, phomaligol A1, phomaligol A hydroperoxide, and phomaligol A1 hydroperoxide were previously isolated from *Phoma lingam* and *Phoma wasabiae*, but their biological activity has not been reported yet [47,48].

### 3.5. Pigment

**Figure 7:**
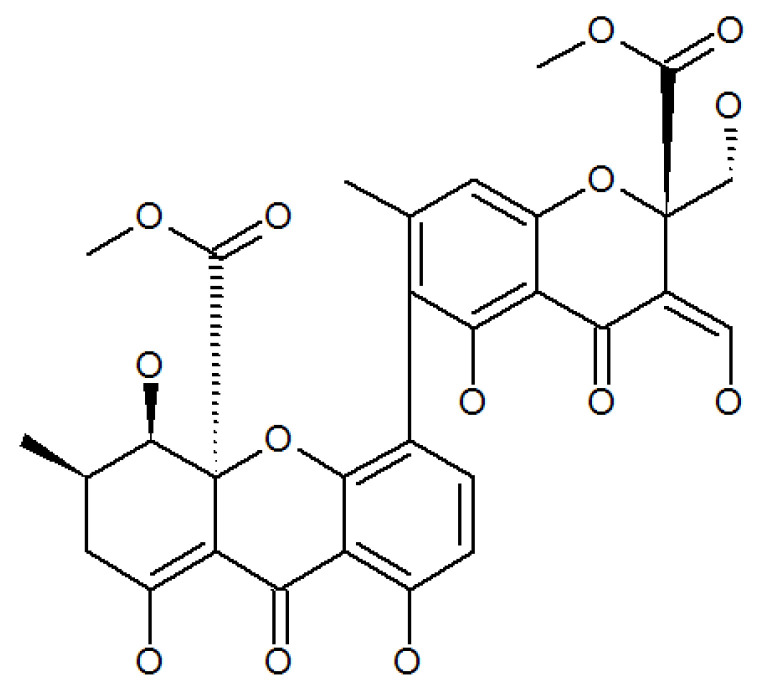
The structure of purpureone.

Lenta investigated the ethyl acetate extract of the mycelium of *P. lilacinum* isolated from the pigment purpureone (Figure 7, Table 1), which was found to possess potent antileishmanial activity and antibacterial activity [49].

## 4. Other Compounds

### 4.1. Ergosterols

**Figure 8:**
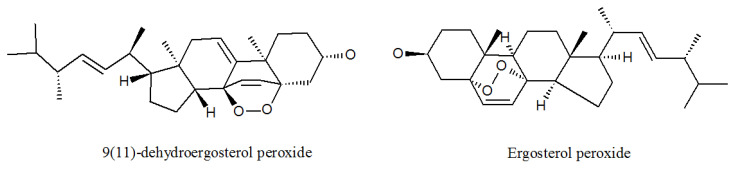
The structures of ergosterols.

Professor Cui and his team isolated seven compounds from the metabolites of *P. lilacinum* ZBY-1 identified as 9(11)-dehydroergosterol peroxide, ergosterol peroxide (Figure 8, Table 1), (22E,24R)-5α,6α-epoxy-3β-hydroxyergosta-22-ene-7-one, and cerebrosides A, B, C, and D. 9(11)-dehydroergosterol peroxide, ergosterol peroxide, and (22E,24R)-5α,6α-epoxy-3β-hydroxyergosta-22-ene-7-one had the inhibitory effect of human cancer K562, MCF-7, HL-60, and BGC-823 cells, with their IC_50_ values on these cell lines ranging from 9.5 mg/L to 59.6 mg/L [50]. 9(11)-dehydroergosterol peroxide and ergosterol peroxide were found to be useful for developing a therapeutic candidate for lung cancer complications [51,52]. These three compounds all show high medical value and provide good materials for the research and development of anti-cancer drugs.

### 4.2. Cerebrosides

**Figure 9:**
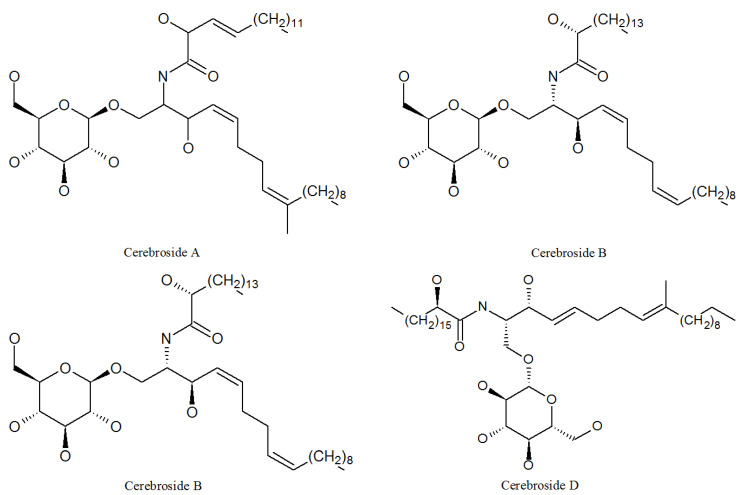
The structures of cerebrosides.

Cerebrosides A, B, C, and D (Figure 9, Table 1) were isolated from the *P. lilacinum* ZBY-1 strain by professor Cui in 2013, and they are all linear analogs. Cerebrosides function as a non-race-specific elicitor in a wide range of plant-phytopathogenic fungus interactions [53]; cerebrosides A and B have significant effects of analgesia and brain protection [54]. Cerebrosides C and D were known to potentiate the activity of cell wall-active antibiotics [55]. In addition, cerebrosides A and C play a role in cell growth, differentiation, and apoptosis in animals [56].

### 4.3. Paecilaminols and Others

**Figure 10:**
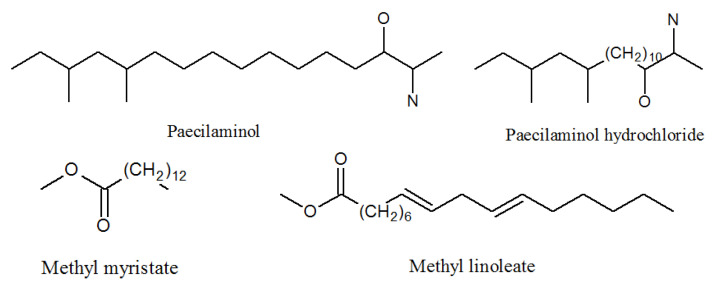

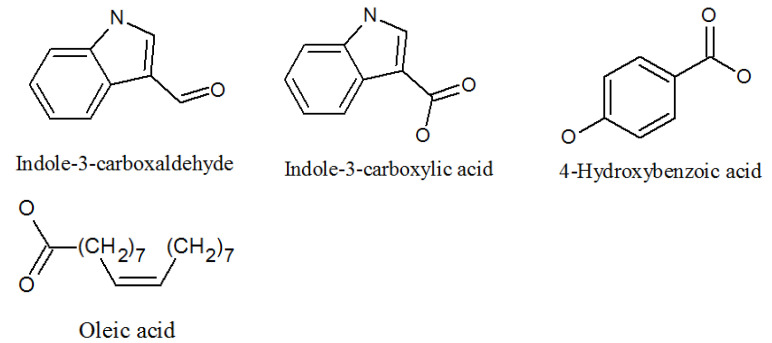
The structures of paecilaminols and others.

Cui found 11 compounds in the year of 2012 identified as paecilaminol, paecilaminol hydrochloride, 1(2)-linolyl-2(1)-palmityl-glycero-*O*-4′-(*N*,*N*,*N*-trimethyl) homoserine, 1,2-dilinolylglycero-*O*-4′-(*N*,*N*,*N*-trimethyl) homoserine, Me myristate, Me linoleate, linoleate, oleic acid, indole-3-carboxaldehyde, indolyl-3-carboxylic acid, and 4-hydroxybenzoic acid (Figure 10, Table 1). Compounds paecilaminol and paecilaminol hydrochloride have the ability to inhibit human cancer K562, MCF-7, HL-60, and BGC-823 cells. Compound paecilaminol was a major antitumor metabolite of the strain *P. lilacinum* ZBY-1 and it is also a NADH-fumarate reductase inhibitor [57].

Me myristate was promised to be exploited as a hydrophobic medical carrier [58]. Me linoleate exhibited cytotoxic antibacterial activities against *Bacillus subtilis* and *Staphylococcus aureus* [59,60]. Modern medicine often makes acute respiratory distress syndrome models by injecting oleic acid into rabbits or rats [61].

Indole-3-carboxaldehyde has useful antimicrobial properties, and it can inhibit the atopic dermatitis-like inflammation induced by MC903 [62,63]. Hydroxybenzoic acids such as 4-hydroxybenzoic acid and 3,4-dihydroxybenzoic acid have various functional biological properties, including anticancer, antibacterial, antiaging, antidiabetic, anti-inflammatory, and antiviral activities. They are widely used in food, cosmetic, and pharmaceutical industries [64]. In addition, 4-hydroxybenzoic acid increased after an organic intervention diet, and it may help with health-promoting qualities in the near future [65,66].

**Table 1:** SMs isolated from *Purpureocillium lilacinum* and their biological activities.

Metabolites	CAS. No	Material Source	Biological Activity
Leucinostatin A	76600-38-9	*P. lilacinus* ZBY-1 from deep sea water	Inhibited prostate cancer cells [29], nematocidal activity [26], activity against Gram-positive bacteria [27].
Leucinostatin B	159544-15-7	Culture medium of *P. lilacinm*	Treatment of systemic candidiasis, nematocidal activity [26], activity against Gram-positive bacteria [27].
Leucinostatin C	110483-88-0	Culture medium of *P. lilacinm*	Drug-related side-effects and adverse reactions activity against Gram-positive bacteria [27], nematocidal activity [26].
Leucinostatin D	100334-47-2	Cultivated, mycelia complex of *P. marquandii*	Activity against Gram-positive bacteria [27], nematocidal activity [26].
Leucinostatin F		Culture medium of *P. lilacinm*	Unknown
Leucinostatin H	109539-58-4	Culture medium of *P. lilacinm*	Nematocidal activity [26].
Leucinostatin K	109539-57-3	Culture medium of *P. lilacinm*	Nematocidal activity [26].
Leucinostatin Y		Mycelia, cultivated complex of *P. linacinus* 40-H-28	Preferential cytotoxicity to cancer cells under glucose-deprived conditions and inhibition of mitochondrial function [32].
Acremoxanthone C	1360445-63-1P	Cultivated, mycelia complex of *P. lilacinm*	Cytotoxicity and 20 s proteasome inhibitory activity; high affinity with human calmodulin biosensors [37]; anti-oomycete activities [38]; exhibited anti-*Bacillus cereus*, antibacterial, antifungal, antiplasmodial, and cytotoxic activity; Gram-positive bacteria [36].
Acremoxanthone D	1360445-62-0P	Cultivated, mycelia complex of *P. lilacinm*	Moderate 20 s proteasome inhibitory activity [37].
Acremoxanthone F	1882150-25-5P	Cultivated, mycelia complex of *P. lilacinm*	Antimalarial activity against plasmodium falciparum K1 strain and multidrug-resistant strain [39].
Acremoxanthone G	1882150-26-6P	Cultivated, mycelia complex of *P. lilacinm*	Antimalarial activity against plasmodium falciparum K1 strain and multidrug-resistant strain [39].
Acremonidin A	701914-77-4P	Cultivated, mycelia complex of *P. lilacinm*	Moderate activity Against Gram-positive bacteria [36].
Acremonidin C	701914-79-6P	Cultivated, mycelia complex of *P. lilacinm*	Antibacterial activity [36].
Acremonidin G	1882150-23-3P	*P. lilacinus* ZBY-1 from deep sea water	Anti-enterococcus faecium activity [39].
Paecilomide	1538575-22-2P	Cultivated, mycelia complex of *P. lilacinm*	Acetylcholinesterase inhibitor [41].
9(11)-dehydroergosterolperoxide	91579717	*P. lilacinus* ZBY-1 from deep sea water	Cytotoxic effect [51].
Ergosterol peroxide	2061-64-5	*P. lilacinus* ZBY-1 from deep sea water	Exhibits antimycobacterial, trypanocidal, and antineoplastic activities [51].
(22E,24R)-5α, 6α-epoxy-3β-hydroxyergosta-22-ene-7-one		*P. lilacinus* ZBY-1 from deep sea water	Inhibitory effect of human cancer K562, MCF-7, HL-60, and BGC-823 cells [50].
Cerebroside A	115681-40-8	*P. lilacinus* ZBY-1 from deep sea water	Induction of cell growth, differentiation, and apoptosis in animals [56].
Cerebroside B	88642-46-0	*P. lilacinus* ZBY-1 from deep sea water	Causes disease such as fusariosis, colitis, and apnea
Cerebroside C	98677-33-9	*P. lilacinus* ZBY-1 from deep sea water	Activity of cell wall-active; antibiotics; induction of cell growth, differentiation, and apoptosis in animals [55].
Cerebroside D	113773-89-0	*P. lilacinus* ZBY-1 from deep sea water	Activity of cell wall-active antibiotics [55].
Paecilopyrone A	1173292-70-0	Cultivated, mycelia complex of *P. lilacinm*	Unknown
Paecilopyrone B	1173292-71-1	Same as above	Unknown
Phomapyrone B	157744-25-7	Same as above	Unknown
Micropyrone	54682570	Same as above	Unknown
Phomapyrone C	157744-26-8	Same as above	Unknown
Kojic acid	501-30-4	Same as above	Antibacterial activities; tyrosinase inhibitory activity [44].
Phomaligol A	152204-32-5	Same as above	Unknown
Phomaligol A1	152053-11-7	Same as above	Unknown
Methylphomaligol A	152159-01-8	Same as above	Unknown
Acetylphomaligol A	1173292-72-2	Same as above	Unknown
Phomaligol A hydroperoxide	181798-75-4	Same as above	Unknown
Phomaligol A1 hydroperoxide	182072-72-6	Same as above	Unknown
Phomaligol B	1173292-73-3	Same as above	Unknown
Phomaligol C	1173292-74-4	Same as above	Unknown
Paecilaminol	540770-33-0	Same as above	Inhibits human cancer cell K562, MCF-7, HL-60, and BGC-823 cells [50].
Paecilaminol Hydrochloride	1650570-79-8	Same as above	Inhibits human cancer cell K562, MCF-7, HL-60, and BGC-823 cells
Me myristate	124-10-7	Same as above	Medical carrier [50].
Me linoleate	112-63-0	Same as above	Exhibited cytotoxic antibacterial activities against *Bacillus subtilis* and *Staphylococcus aureus* [59].
Indole-3-carboxaldehyde	487-89-8	Same as above	Antimicrobial properties [62].
Indolyl-3-carboxylic acid	771-50-6	Same as above	Potential in vitro antimalarial, anticancer activity [63].
4-hydroxybenzoic acid	99-96-7	Same as above	Inhibits LPS-induced protein [64].
Purpureone	2231079-10-8P	Mycelium of *P. lilacinm*	Antileishmanial activity; antibacterial activity [49].

## 5. Biosynthesis of Secondary Metabolites in *Purpureocillium lilacinum*

In 2015, Prasad sequenced the TERIBC-1 strain of *P. lilacinus* with a genome size of 40.02 Mb by using Illumina Hiseq technology, and predicted 30 secondary metabolite synthesis genes: 12 polyketide syntheses (PKs, details of all abbreviations are in Table S1), 2 PKs-like, 7 nonribosome peptide synthetases, 7 NRPSs-like, 1 PK-NRPS, and 1 dimethylallyl tryptophan synthases (DMATs) gene [67]. In 2016, Wang sequenced *P. lilacinus* PLBJ-1 and PLFJ-1 strains. The genome sizes of the two strains were 38.14 Mb and 38.53 Mb, respectively [18]. Using SMURF [68] and anti-SMASH [69] software to predict the secondary metabolite synthesis gene cluster, PLBJ-1 and PLFJ-1 strains were found to encode 13 PKs, 2 PKs-like, 10 NRPSs, 10 NRPSs-like, 1 PK-NRPS, 4 terpene synthases (TSs), and 1 DMAT genes. It can be seen that the secondary metabolites produced by different species of *P. lilacinum* are not identical, but in general, *P. lilacinum* has great potential in the synthesis of secondary metabolites.

**Figure 11:**
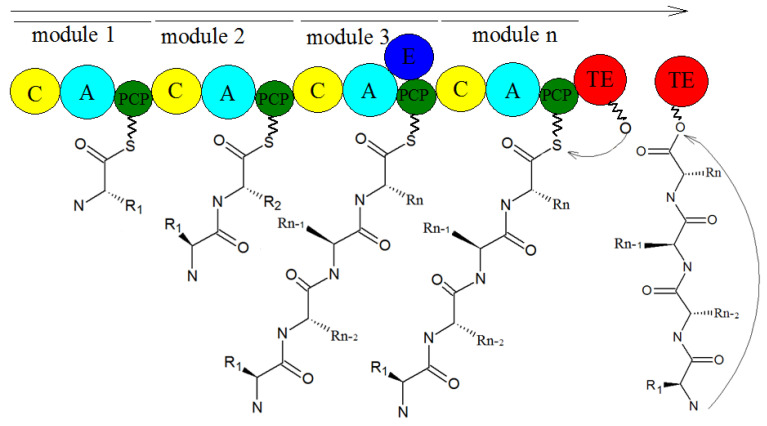
The biosynthesis of NRPSs [76].

We know that the typical NRPS modules have adenylation (A), thiolation (T) or peptidyl carrier (PCP), condensation (C), and thioesterase (TE) domains [70,71], which are, respectively, responsible for the activation of amino acids, the extension of peptide chains, the formation of amide bonds, and the release of peptide chains [72,73]. The synthesis mechanism of NRPSs is shown in Figure 11. Generally speaking, the A domain combines with the amino acid substrate under the action of ATP to form the corresponding aminoacyl AMP, and the aminoacyl AMP combines with the sulfhydryl group of the T domain to form the aminoacyl-s-carrier complex. Finally, the carriers carrying the aminoacyl group and the peptide acyl group combine with the specific region of the C domain, and the amino group on the aminoacyl-s-carrier complex attacks the acyl group on the peptidyl-s-carrier complex, forming a new peptide bond, and finally forming a complete peptide chain, through the action of multiple modules, wherein the amino acids in the peptide chain correspond to the modules in the NRPS one by one. Some NRPS modules also contain epimerization (E), formylation (F), methylation (M), heterocyclization (CY) [74], reduction (R), and oxidation (OX) domains, which are involved in the structural modification of peptide chains. Finally, mature peptide chains are released from the NRP assembly line under the action of the TE domain [75].

In 2016, Wang sequenced and analyzed the whole genome of *P. lilacinum*, and predicted the knock-out of the NRPS synthetic gene (LcsA), PK synthetase (LcsC), Acyl CoA ligase (LcsD), and thioesterase (LcsE), using high-performance liquid chromatography (HPLC) to compare the crude extracts of wild-type and mutant strains of *P. lilacinum*. It was found that the crude extracts of ΔLcsA, ΔLcsC, ΔLcsD, and ΔLcsE had a lack of Leucinostatin A and Leucinostatin B, and then these enzymes were found to play a key role in the synthesis of Leucinostatin, and the synthesis of leucinostatin of *P. lilacinum* was suggested. This hypothetical biosynthesis is initiated by the assembly of 4-methylhex-2-enoic acid via reductive PKs. However, they were unable to estimate which PKs were responsible for 4-methylhex-2-enoic acid [18].

**Figure 12:**
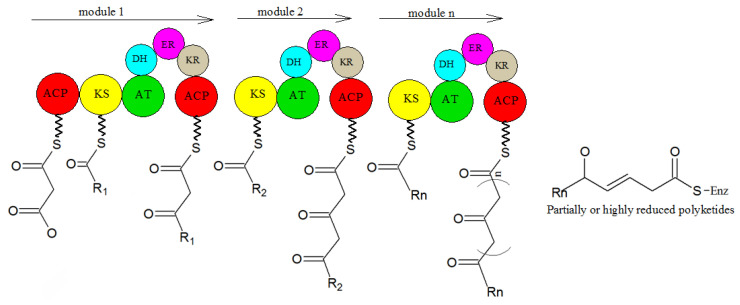
The biosynthesis of PKs [77].

In microorganisms, PK comes from the independent hypothesis of a variety of compounds. Polyketide compounds are assembled by repeated Claisen condensations between the activated acyl initiation unit and the chain extender unit derived from malonyl-CoA. This process is catalyzed by the synergistic action of keto synthase (KS), acyltransferase (AT), and phosphopan ethylation acyl carrier protein (ACP) or CoA linked to the primary chain. After each extension step, the functionality of β-keto can be reduced by further involved enzymes [76]. This general PK catalytic mechanism is realized by different enzyme mechanisms (Figure 12). Three types of PKs are described below, which are responsible for the biosynthesis of polyketide chains.

PKs can be divided into three types: type I PKs are modular enzymes composed of several functional domains, which are arranged linearly and covalently. Any functional domain is not reused in the process of chain synthesis and extension. They mainly synthesize polyether, polyene, and macrolides. PKs of type II are aromatic, starting from acetyl CoA. Polyketones with an aromatic ring structure are synthesized with malonyl coenzyme A as an extension unit. Type III PKs are chalcone synthetases, a kind of homologous dimer enzyme that can be reused. It catalyzes the condensation of acetyl-CoA molecules to synthesize one ring or multi-ring aromatic polyketones [74].

It is generally believed that most α-pyrones are synthesized through the polyketide pathway [78]. Terpenoids are a kind of chain or cyclic secondary metabolites, which are composed of isoprene as the basic unit. Terpenoids are synthesized by terpene synthase and can be divided into: monoterpenes, with geranyl diphosphate as the synthetic precursor; sesquiterpenes, with farnesyl diphosphate as the synthetic precursor; diterpenes, with geranyl pyrophosphate as the precursor. According to the degree of reduction, it can be divided into reduced terpenoids and nonreduced terpenoids [79].

## 6. Problems and Perspectives

Among the more than 40 metabolites reviewed in this paper, we can see that most SMs of *P. lilacinus* that have been reported so far have the functions of anticancer activity, antimicrobial activity, insecticidal activity, cytotoxicity, drug carriers, and so on. Most importantly, some of the compounds showed potent activities compared to those of the positive controls, which indicates that they could be used to develop new medicines. These include the anticancer lead compound leucinostatins, ergosterol peroxide, (22E,24R)-5α, 6α-epoxy-3β-hydroxyergosta-22-ene-7-one, and paecilaminol. Leucinostatins is cytotoxic to HeLa cells, Ehrlich subcutaneous solid tumors, and prostate cancer. However, it was found to be toxic to rats by intraperitoneal injection, so more attention should be paid to its safety assessment when developing the drug. The other three compounds have the ability to inhibit human cancer K562, MCF-7, HL-60, and BGC-823 cells, but their safety for other species is still unknown. Acremoxanthone and acremonidin were both calmodulin inhibitors; paecilomide is an acetylcholinesterase inhibitor and kojic acid showed tyrosinase inhibitory activity, indicating their potential as insecticides. These remarkable activities make many of these compounds suitable candidates for new drugs and insecticides discovery and may lead to future synthesis studies. However, some of the SMs of *P. lilacinus* are toxic to animals and humanity. Hocquette, Dr. Qian, Pastor, and others have reported infections caused by *P. lilacinus* in immunocompromised patients [80,81].

With the development of society, more and more attention has been paid to biological control, more and more fungal products will come out, and the safety of related products has also received great attention. Therefore, how to ensure the safety of fungal products has become particularly important.

Generally, in production and in life, there are six destinations (i.e., target organisms, nontarget organisms, soil, water, atmosphere, and humans) involved in the production and application of *P. lilacinum* pesticide formulations. The most important destination is target organisms, including pests and crops when preparations are released in fields. Soil is another important destination, especially when it is released through soil treatments for nematodes. Water and the atmosphere are the destinations of the drifting formulations. Humans contact *P. lilacinum* through direct and indirect pathways. There is no doubt that the biosafety risks of *P. lilacinum* are closely related to the sources and fates of the SMs produced by entomopathogenic fungi [24].

Surveying the SMs will be beneficial to improving the safety of *P. lilacinum* fungal products. Thus, developing the discovery, structure, function, and synthesis pathway of secondary metabolites of *P. lilacinum* are of great significance to biomedicine, human health, and agricultural disease control. For a long time, due to the gene silencing or low expression of most gene clusters in common culture medium, the research of fungal secondary metabolites has been hindered to some extent. There are only a few kinds of research on SMs of *P. lilacinum*, which are leucinostatins, acremoxanthones, and paecilomides, and their synthetic pathway and regulatory mechanism are still unclear. Therefore, it is necessary to use a super-expressing transcription factor, to replace the promoter in the synthetic gene cluster with an inducible strong promoter, to modify the histone, to heterologously express the gene cluster to activate the silent gene cluster, and to further discover that the structure is novel and biologically active. The SMs production yield of *P. lilacinum* needs to be improved by changing the culture conditions. First, gene knockout methods need to be used to further clarify the synthesis mechanism of secondary metabolites. In addition, it is necessary to continuously improve the efficiency and precision of chemical separation detection, in order to be more conducive to the separation of secondary metabolites and the identification of structural functions.

## Data Availability

Not applicable.

## Supplementary Materials

The following are available online. Table S1: Information of all abbreviations.
