# Supplementary material for: Secondary Metabolites of Purpureocillium lilacinum
Source: Molecules. 2021 Dec 21;27(1):18. doi: 10.3390/molecules27010018 (PMC8746413; doi:10.3390/molecules27010018)
Supplement: Supplementary file 1 [file molecules-27-00018-s001.zip › molecules-1498189-supplementary.pdf]

Table S1. Information of all Abbreviations

Abbreviations	Full Name
SMs	Secondary metabolites
PKs	Polyketide syntheses
NRPs	Non ribosome peptide syntheses
DMATs	Dimethylally tryptophan synthases
TS	Terpene synthases
A	Adenylation
T	Thiolation
PCP	Peptidyl carrier
C	Condensation
TE	Thioesterase
E	Epimerization
F	Formylation
M	Methylation
CY	Heterocyclization
R	Reduction
OX	Oxidation
HPLC	High performance liquid chromatography
KS	Keto synthase
AT	Acyltransferase
ACP	Acyl carrier protein
